# The athletic hip. Joint status and muscle strength among the key elements in the return to sport process

**DOI:** 10.3389/fspor.2025.1690282

**Published:** 2025-11-12

**Authors:** Mario Bizzini, Nicola A. Maffiuletti, Michael Leunig

**Affiliations:** 1 Human Performance Lab, Schulthess Clinic, Zürich, Switzerland; 2 Hip Service, Department of Orthopedics, Schulthess Clinic, Zürich, Switzerland

**Keywords:** hip, muscle strength testing, joint status, return to sport, return to performance

## Introduction

Athletes suffering from hip/groin pain or following hip surgery often demonstrate impairments in hip muscle strength, which need to be addressed in the rehabilitation and training process (1, 2). While hip and groin injuries are common in male team sport athletes, recent research has described a similar problem magnitude in female athletes (3, 4). Considering the demands of high-impact sports such as ice hockey and soccer, both morphological hip abnormalities and hip muscle weakness may put athletes at an elevated injury risk (5–7). Therefore, knowledge of mechanical hip/groin pain and hip muscle strength testing procedures are of utmost importance both in clinical practice and research. While hand-held dynamometry has been the classical choice for testing hip muscles, fixed-frame (or stabilized) dynamometers have become extremely popular in the last 10 years (1, 8, 9). The presence of different devices and testing protocols—implying for example diverse body positions and contraction tasks—requires critical thinking about the adoption of a given test methodology and interpretation of the subsequent strength data (10). An individualized, sport-specific return to sport (RTS) progression is paramount in the rehabilitation and training process (1, 11, 12). Functional performance testing further corroborates the longitudinal evaluation of injured athletes towards their higher level of sporting activity (13). In parallel, knowledge about the joint status of the athletic hip should be taken into the equation when guiding the RTS and, ultimately, the return to competition process (5, 14) on an individual basis. The biological, psychological and social factors influencing the athlete's return to sport should be considered in the context of shared decision-making between all stakeholders (15).

The aim of this opinion paper is to provide a current update on the athletic hip, focusing on the role of hip joint health and hip muscle strength testing in the rehabilitation and RTS process.

## The athletic hip

Clinicians need to recognize the considerable inter-subject differences when facing hip patients/athletes. Besides age and sex, these individuals differ substantially in terms of status and level of activity (recreational, amateur, sub-elite, elite, professional), which has huge implications in term of management during rehabilitation, training and RTS. In parallel, knowledge about the sport- and individual-specific training and competition demands is also crucial. When dealing with an injury (in this case: hip joint problems), specific knowledge about the medical status and recovery of hip joint and muscle function is particularly important.

During the last 15 years, groin pain in athletes has been better understood, for example with the differentiation between adductor-, iliopsoas-, inguinal-, and pubic-related groin pain (1). Accordingly, evidence-based management of athletes with groin problems has been proposed (1). As an example, because hip adduction strength is reduced in soccer players with adductor-related groin pain, a targeted strengthening program has been advocated (1, 7). However, clinicians and researchers have also identified the hip joint as a frequent source of hip/groin problems (hip-related groin pain or hip-related pain). Bedi et al. have proposed a differentiation between dynamic and static mechanical causes of hip joint pain (5). In athletes with femoroacetabular impingement syndrome (FAIS)—for example, a young male ice hockey player—the biomechanics of the hip joint is altered due to the cam or pincer morphology, causing abnormal stress and contact between the femoral head and acetabular rim during repetitive hip motions. In turn, these changes have been shown to modify the dynamic muscle forces around the pelvis, which may lead to pain/symptoms—often localized in the anteromedial groin region—and hip muscle weakness (5). The most common muscles affected by dynamic impingement are the adductor longus, proximal hamstrings, hip abductors and hip flexors (5). In athletes with an underlying hip joint dysplasia—for example, a young female ballet dancer—static factors may cause abnormal stress and asymmetric loading across the hip joint, leading to chondral wear aces with or without associated hip joint instability. Athletes with hip static overload may also experience anteromedial groin pain, whereas compensatory muscle weakness—affecting in particular hip abductors, hip adductors and the iliopsoas—can provoke muscle fatigue and pain around the hip and pelvis (5).

The “2016 Consensus Statement on Return to Sport” represents an excellent framework for clinicians and stakeholders. According to this consensus statement, the RTS process should be seen as a continuum comprising three elements: return to participation, RTS and return to performance (15). The “ultimate goal” for athletes would be to reach their pre-injury level in terms of training and performance, in an optimal physiological and psychological condition. Considering the biopsychological model of RTS after injury/surgery, clinicians, stakeholders and even the athlete/patient should be aware that this “ultimate goal” cannot always be achieved, and that even a “removal from sport” should be considered (15). The primary goal of the medical team is and should always be to protect the health of the athlete both in the short and long term.

## Strength testing during the rehabilitation process

**Figure 1:**
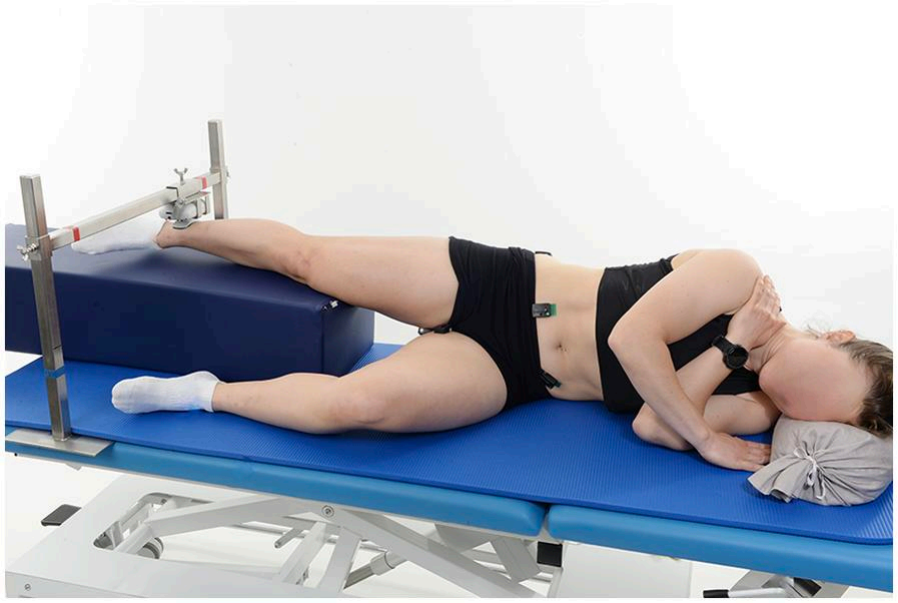
Setting for right hip abduction strength test.

While respecting pain/symptoms, it is crucial to integrate exercises for the different hip muscle groups early in the rehabilitation program. Both isolated hip exercises (i.e., flexion/extension, abduction/adduction, internal/external rotation) and multi-segment exercises (such as squats, lunges, planks) should be part of the strengthening protocol for the injured athletic hip (11, 12). As an example, hip adduction strength is often reduced in male athletes with groin pain (1), while hip flexor, abductor and adductor strength deficits have been reported in male and female athletes with FAIS (1, 16). Strength testing of isolated hip muscles—ideally in all planes of motion—should therefore represent a key element in the rehabilitation/training process. Muscle-specific weakness may go undetected when evaluating strength using multi-segment exercises only (17). The use of hand-held dynamometers (HHD) and fixed-frame systems (FFS)—though not interchangeable—has been proven to be both valid and reliable for the assessment of hip muscle strength (18), while offering a portable and cost-effective solution compared to isokinetic dynamometry. Recent research has shown that the type of contraction task (unilateral vs. bilateral) and body position (e.g., supine vs. side-lying) can greatly influence the validity of hip strength assessments, hip adductors/abductors in particular. As an example, during bilateral testing of hip adductors and abductors in the supine position (a popular setting with FFS), strength values were extremely similar for the left and right side (and bilaterally “facilitated” due to Newton's action-reaction law), leading to a significant underestimation of interlimb asymmetries (10). On the other hand, unilateral hip adductor strength testing in the same position resulted in lower agonist muscle activity and higher stabilizer activity of the contralateral obliqui abdominis compared to bilateral testing (10). Therefore, the side-lying position should be preferred for hip adductor/abductor strength testing (see Figure 1), as it offers the most valid and reliable setup (19).

## Functional testing during the RTS process

A recent international consensus highlighted the need of integrating validated functional performance tests—such as for neuromuscular control, muscle strength, etc.—in athletes dealing with hip problems (13). Tests such as the star excursion balance test and variants are classified as medium-intensity with low impact and controlled speed. High-intensity tests, such as the different hop and agility tests, are multiplanar with high impact and fast speeds (13). Neurocognitive components have been increasingly added to these classical functional performance tests (20), with the goal to improve their ecological validity. This emerging research area highlights the need of multisensory integration and cognitive processing demands both in rehabilitation/training and testing (20).

Several functional performance tests require high levels of hip muscle strength. Therefore, parallel to general strengthening, training of selected hip muscle groups should be continued in the later stages of rehabilitation, as deficits may persist for several months after injury/surgery (21). The absence of pain/symptoms in the hip/groin areas is also crucial to enable the athlete to successfully regain strength, as pain has a substantial influence on the voluntary force generating capacity of the major hip muscles (22).

The use of normative hip muscle strength data but also ratios (e.g., involved/uninvolved, adductor/abductor) has to be considered with caution, as these data often vary with age and sex as well as between sports, level of practice, playing position and limb dominance. Such data exist for relatively small samples of ice hockey, soccer, and Australian football players (7, 8, 9), mainly for hip abduction/adduction strength. Pre-injury/surgery data should ideally be used for longitudinal comparisons with post-injury/surgery data, allowing for an optimal appraisal of hip muscle strength recovery at the individual level.

## Performance-based sport-specific testing

According to the recent international consensus (13), sport-specific tests should mimic the reactive and neurocognitive demands and the decision-making of real sport situations. This means that specific training drills should be used as test situations, allowing for an estimation of the athlete's ability to withstand the specific demands of the sport (23). However, validated sport-specific tests to assess readiness for full team training are still lacking, especially in post-injury or post-surgical contexts. For football/soccer players, the Yo-Yo Intermittent Recovery Test and the Repeated-Shuttle-Sprint-Ability Test are considered the best performance tests in terms of validity and reliability (23). For ice hockey players, a battery of agility and speed tests is available (24). The availability of pre-injury/surgery data—together with GPS data collected during training and competition—offer individual tracking of the athlete status, thus allowing objective monitoring of the training loads. Together, this may help to identify when an athlete is able to train/perform at pre-injury/surgery levels (25).

Psychological readiness, as largely documented in the field of ACL injury/surgery (15), plays an important role in the return to sport/competition process also for athletes after hip injury/surgery. Recently, the Hip-Return to Sport after Injury scale was developed for the assessment of psychological readiness in hip arthroscopy patients (26). Interestingly, patients/athletes who returned to a high level of performance following hip arthroscopy had higher scores compared to others who returned to lower sport activity levels (26). However, psychological recovery and physiological recovery following hip injury/surgery are two different constructs, and their relationships in the RTS process are still not well understood (27).

## Should the hip joint status play a role in RTS decision-making?

Athletes undergoing hip preservation surgery for FAIS should be aware that the presence of degenerative or cartilage damage is a potential risk factor for further damage to the hip, especially in case of high-impact sports. While recreational athletes may transition to lower impact sports after hip surgery, professional athletes often do not want or cannot reduce their activity level due to various reasons (financial/career issues) (15, 28). It has been found that increased age and clinical factors linked with degenerative joint disease were associated with the need for total hip arthroplasty within 1–8 years following hip arthroscopy (29). Therefore, it is the duty of the medical team to discuss the hip joint's health, and to provide sound information and realistic expectations towards the athlete's RTS goals.

## Final considerations

While previous publications (mostly cases studies, unspecific definition) showed high RTS rates following hip surgery, recent research (cross-sectional design, stricter definition) have indicated that “only” 50% of FAIS patients/athletes were able to return to their pre-injury sport level, and only 20% to their previous level of performance (21). Following hip arthroscopy, most FAIS athletes with impaired performance experience difficulties in high-speed running and during explosive movements (30). Recreational athletes often have lower RTS rates when compared with elite athletes, also because they are more prone to change or even discontinue their previous sport (28). In a 15-year study conducted with elite football players, 11% of all hip/groin (time-loss) injuries were re-injuries, indicating the importance of secondary/tertiary prevention in hip injury/surgery athletes (31). These RTS and recurrence rates suggest that rehabilitation and training programs should be further optimized. Specific strengthening and stabilization exercises should be performed regularly as a routine pre-training warm up, and/or with dedicated sessions integrated into the training plan. There is also a need to consider more valid/reliable hip muscle strength and functional performance tests during the rehabilitation and training process. A comprehensive approach to hip joint health highlights the importance of individualized treatment and RTS strategies—including hip muscle strength testing—for athletes experiencing hip-related pain.
